# Genome Sequences of Three Salmonella enterica subsp. enterica Serovar Infantis Strains from Healthy Broiler Chicks in Hungary and in the United Kingdom

**DOI:** 10.1128/genomeA.01468-14

**Published:** 2015-02-12

**Authors:** Ferenc Olasz, Tibor Nagy, Móni Szabó, János Kiss, Ama Szmolka, Endre Barta, Andries van Tonder, Nicholas Thomson, Paul Barrow, Béla Nagy

**Affiliations:** a Agricultural Biotechnology Institute of the National Agricultural Research and Innovation Centre, Gödöllő, Hungary; b Institute for Veterinary Medical Research, Centre for Agricultural Research, Hungarian Academy of Sciences, Budapest, Hungary; c Nuffield Department of Medicine, University of Oxford, Peter Medawar Building for Pathogen Research, Oxford, United Kingdom; d Wellcome Trust Sanger Institute, Wellcome Trust Genome Campus, Hinxton, Cambridge, United Kingdom; e School of Veterinary Medicine and Science, University of Nottingham, Sutton Bonington, Leicestershire, United Kingdom

## Abstract

The genome sequences of three strains of Salmonella enterica subsp. enterica serovar Infantis isolated from broiler chickens in 1994 and 2004 in Hungary and in the 1980s in the United Kingdom are reported here. A sequence comparison should improve our understanding of the evolution of the genome and spread of *S.* Infantis in poultry.

## GENOME ANNOUNCEMENT

Salmonella enterica subsp. enterica serovar Infantis spreads among broilers in Hungary (1) and elsewhere in Europe but not in the United Kingdom (2). Here, we present the genome sequences of three *Salmonella* Infantis strains isolated from chickens from the 1980s to the 2000s.

Fragment libraries of 500 bp were prepared from the total DNA of strains SI 69/94 and SI 54/04 (Hungary), and the 2 × 100-bp Illumina paired-end genome sequencing was performed by Aros Applied Biotechnology (Aarhus, Denmark) as a custom service, using the Illumina HiSeq 2000 platform. The numbers of reads are 34.4 million for SI 69/94 and 95 million for SI 54/04. The estimated coverages of the whole genome are 600× and 1,700×, respectively. The whole genome of *S.* Infantis strain 1326/28 (United Kingdom) (3, 4), sequenced to a coverage depth of 9× from M13mp18 (insert size, 1.4 to 2 kb) and pUC18 (insert size, 2.2 to 4.2 kb) small-insert libraries using BigDye Terminator chemistry on ABI3700 automated sequencers. The end sequences from a larger-insert plasmid (pBACe3.6, 12- to 30-kb insert size) libraries were used as a scaffold. The sequence was assembled, finished, and annotated, as described previously (5), using the program Artemis (6) to collate data and facilitate annotation.

The estimated coverage of the subsets of reads was adjusted to 30× for both Hungarian strains, and these were *de novo* assembled using Mira version 3.9.15 (7). The G+C content appeared to be same for all three strains (52.29%). The draft sequence of strain SI 54/04 contains additional scaffolds that cannot be aligned to those of SI 69/94 and the chromosome of 1326/28, suggesting the presence of a ca. 277-kbp plasmid in strain SI 54/04.

Randomly selected subsets of reads were also applied for the reference genome assisted assembly, using the assembled United Kingdom strain 1326/28 as a reference backbone. The chromosome lengths of strains 1326/28, SI 69/94, and SI 54/04 have been determined to be 4,710,675, 4,710,832, and 4,710,839 bp, respectively.

The assembled genome sequences were submitted to the RAST annotation server (8). We set the taxon to S. enterica, the genetic code to 11 (*Archaea*, *Bacteria*), the sequence method to “other,” the coverage to >8×, and “automatically fix errors” to “no.” For SI 69/94, SI 54/04, and 1326/28, we obtained the following data: 4,664, 4,623, and 4,671 annotated genes, respectively; 170, 172, and 168 tRNAs, respectively; and 44 rRNAs in all three strains.

A comparison of the chromosomal nucleotide sequences revealed that the three strains exhibit marked similarity (minimum, 99.94%; maximum, 99.97%). Comparing 1326/28 to the published Israeli strains of human origin (9), 119944 (GenBank assembly ID GCA_000506925.1) and 335-3 (GenBank assembly ID, GCA_000506945.1), 92.63% and 100% similarities were observed, respectively. The genome sequences of the three *S.* Infantis strains revealed a strikingly limited similarity (about 83%) to the SARB27 sequences (GenBank accession no. CM001274), identified earlier as *S.* Infantis (10), but indicating an unusual evolutionary distance.

### Nucleotide sequence accession numbers.

The draft genome sequences of SI 69/94 and SI 54/04 and the assembled and annotated genome sequence of 1326/28 have been deposited in the NCBI GenBank database under the accession numbers JRXB00000000, JRXC00000000, and LN649235, respectively.
